# Tropical Ferns: The Forgotten Source of Treatments for Skin Diseases

**Published:** 2024-07-22

**Authors:** Pablo Jimenez-Bonilla, Alexander Rojas-Alvarado, Victor Alvarez-Valverde

**Affiliations:** 1 Laboratory of Phytochemistry, Chemistry school, Universidad Nacional, Heredia, Costa Rica; 2 Laboratory of Natural Products and Biological Assays, Chemistry school, Universidad Nacional, Heredia, Costa Rica; 3 Department of Chemical Sciences, Universidad Estatal A Distancia, Sabanilla, Costa Rica; 4 School of Biological Sciences, Universidad Nacional, Heredia Costa Rica

## DESCRIPTION

Over the past four decades, various commercial products derived from the Kallawalla fern have been developed and marketed. These products range from oral formulations like syrups and capsules to topical applications like sunscreens. They are widely used for skin photoprotection, treating psoriasis, reducing skin inflammation and addressing conditions like eczema, melasma, atopic dermatitis, photoaging, skin cancer, actinic keratosis, polymorphous light eruption, solar urticaria and vitiligo. Interestingly, despite the passage of time, these natural remedies remain significant in the market due to the limited availability of alternative effective treatments for some of these conditions. The therapy has been considered significant during clinical trials. However, bioactive molecules have not been isolated and the mechanism of action is not completely understood. Furthermore, the mechanisms of action in ferns like Kallawalla are likely due to a combination of multiple molecules rather than a single compound. For instance, in photoprotection, no single molecule can absorb radiation across the entire ultraviolet spectrum. Instead, the collective action of various molecules contributes to the overall protective effect, allowing ferns to effectively shield themselves from a broad range of UV radiation.

Kallawalla is a fern native to Central America, historically referred to as *Polypodium leucotomos*. It is synonymous with *Phlebodium pseudoaureum* (sensu estricto) and *Phlebodium aureum* (sensu lato). Ferns, among the most ancient plant species, possess a primitive and fascinating metabolism. This unique metabolic system allows them to produce a variety of idiolites (secondary metabolites specific for a species or group), which sets them apart from many other organisms. Ferns, including Kallawalla, are notable for their ability to produce antioxidant compounds, primarily phenolic compounds. These antioxidants protect cells from damage caused by free radicals and oxidative stress. Many other ferns related or not to kallawalla are potential sources of bioactive compounds to be used as treatments for skin disorders and other diseases.

Consequently, many species within the Polypodiaceae family exhibit significant potential for use in pharmaceutical applications. Despite their promising attributes, these species have been relatively under-researched. Their unique biochemical properties, including their diverse array of secondary metabolites and antioxidant capabilities, suggest they could play a valuable role in the development of new medicinal preparations. Recently, we identified 17 fern species with a higher content of total phenolic compounds than the roots of *Phlebodium pseudoaureum*. Many of these ferns also exhibit strong photoprotective and antibacterial properties, highlighting their potential for pharmaceutical skin disorders and other diseases.

Significant research is still required to fully understand tropical ferns, particularly in areas such as their mechanisms of action, chemical compositions and complete metabolic pathways. Further investigation is needed into their stability, potential toxicity when consumed orally and the stability of their compounds. This continued research is essential to unlock their full potential for pharmaceutical and other applications. For example, commercial extracts of *P. pseudoaureum* are typically standardized based on their content of quinic acid and total phenolic compounds. However, there is not a clear correlation between these standardized values and the extract’s associated bioactivity. The complex nature of skin diseases suggests that the bioactive effects may result from a complex interaction or the summative effect of various components rather than just the levels of quinic acid and phenolic compounds alone. This approach contrasts with traditional natural products research, which often focuses on identifying single active compounds responsible for a product’s effects, such as allosteric or competitive inhibitors, or activators for a specific enzymatic site. In those cases, a specific molecular structure is usually identified as the primary active ingredient.

Also, ferns hold promise for addressing several pressing issues. Their antimicrobial properties can provide alternative treatments to traditional antibiotics, helping combat the growing problem of antibiotic resistance, which is becoming increasingly widespread. In addition, many personal and skincare products contain molecules that are environmentally persistent. These substances often go undetected by standard wastewater treatment processes, leading to their discharge into natural water bodies, where they disrupt the microbial balance and negatively impact corals and marine life. Natural products derived from ferns may offer a more sustainable solution. While further research is necessary, these natural compounds are less likely to cause environmental toxicity, making them a potentially safer alternative for both humans and the environment.

## CONCLUSION

Finally, there are several characteristics of ferns that require further attention. Many wild fern species are not yet domesticated and can be challenging to cultivate in controlled environments like greenhouses. Some ferns grow individually on rocks, forming sparse populations rather than dense clusters, making them difficult to gather in significant numbers. Additionally, many ferns struggle to adapt to artificial substrates used in greenhouses or have complex reproductive cycles that hinder efficient cultivation. For instance, we’ve observed that certain species exhibit higher greenhouse reproduction rates and levels of quinic and chlorogenic acid, respecting *P. pseudoaureum*. These challenges highlight the need for innovative cultivation methods and more research into the optimal conditions for growing these valuable plants. Also, the adaptation and domestication can create some challenges to optimize the metabolite levels.

